# Individual Brain Metabolic Connectome Indicator Based on Jensen-Shannon Divergence Similarity Estimation Predicts Seizure Outcomes of Temporal Lobe Epilepsy

**DOI:** 10.3389/fcell.2021.803800

**Published:** 2022-01-11

**Authors:** Zehua Zhu, Zhimin Zhang, Xin Gao, Li Feng, Dengming Chen, Zhiquan Yang, Shuo Hu

**Affiliations:** ^1^ Department of Nuclear Medicine, XiangYa Hospital, Changsha, China; ^2^ Department of Blood Transfusion, XiangYa Hospital, Changsha, China; ^3^ Shanghai Universal Medical Imaging Diagnostic Center, Shanghai, China; ^4^ Department of Neurology, Xiangya Hospital, Central South University, Changsha, China; ^5^ Department of Neurosurgery, Xiangya Hospital, Central South University, Changsha, China; ^6^ Key Laboratory of Biological Nanotechnology of National Health Commission, Xiangya Hospital, Central South University, Changsha, China

**Keywords:** PET, FDG-PET, metabolic connectome, temporal lobe epilepsy, predicts seizure outcomes

## Abstract

**Objective:** We aimed to use an individual metabolic connectome method, the Jensen-Shannon Divergence Similarity Estimation (JSSE), to characterize the aberrant connectivity patterns and topological alterations of the individual-level brain metabolic connectome and predict the long-term surgical outcomes in temporal lobe epilepsy (TLE).

**Methods:** A total of 128 patients with TLE (63 females, 65 males; 25.07 ± 12.01 years) who underwent Positron emission tomography (PET) with 18F-fluorodeoxyglucose (FDG) imaging were enrolled. Patients were classified either as experiencing seizure recurrence (SZR) or seizure free (SZF) at least 1 year after surgery. Each individual’s metabolic brain network was ascertained using the proposed JSSE method. We compared the similarity and difference in the JSSE network and its topological measurements between the two groups. The two groups were then classified by combining the information from connection and topological metrics, which was conducted by the multiple kernel support vector machine. The validation was performed using the nested leave-one-out cross-validation strategy to confirm the performance of the methods.

**Results:** With a median follow-up of 33 months, 50% of patients achieved SZF. No relevant differences in clinical features were found between the two groups except age at onset. The proposed JSSE method showed marked degree reductions in IFGoperc.R, ROL. R, IPL. R, and SMG. R; and betweenness reductions in ORBsup.R and IOG. R; meanwhile, it found increases in the degree analysis of CAL. L and PCL. L, and in the betweenness analysis of PreCG.R, IOG. R, PoCG.R, PCL. L and PCL.R. Exploring consensus significant metabolic connections, we observed that the most involved metabolic motor networks were the INS-TPOmid.L, MTG. R-SMG. R, and MTG. R-IPL.R pathways between the two groups, and yielded another detailed individual pathological connectivity in the PHG. R-CAU.L, PHG. R-HIP.L, TPOmid.L-LING.R, TPOmid.L-DCG.R, MOG. R-MTG.R, MOG. R-ANG.R, and IPL. R-IFGoperc.L pathways. These aberrant functional network measures exhibited ideal classification performance in predicting SZF individuals from SZR ones at a sensitivity of 75.00%, a specificity of 92.79%, and an accuracy of 83.59%.

**Conclusion:** The JSSE method indicator can identify abnormal brain networks in predicting an individual’s long-term surgical outcome of TLE, thus potentially constituting a clinically applicable imaging biomarker. The results highlight the biological meaning of the estimated individual brain metabolic connectome.

## Introduction

In most patients with refractory temporal lobe epilepsy (TLE), surgery has proven to be an effective treatment (Tellez-Zenteno et al., 2005). The goal of epilepsy surgery is to render the patient seizure free. However, not every patient with TLE can achieve this outcome postoperatively, as shown by meta-analysis where the median proportion of long-term seizure-free patients was around 60% (Engel et al., 2003a; Tellez-Zenteno et al., 2005). Patients who continued to experience seizures after surgery were directly associated with an even lower quality of life (Schmidt et al., 2004). Given the state of existing research, it is difficult to find practicable markers that can effectively predict surgical outcomes. Therefore, determining the potential characteristics of the different outcomes of TLE and identifying biological indicators for prediction remain critical needs in the management of each patient (Harroud et al., 2012; Jehi et al., 2015b; Giulioni et al., 2016; West et al., 2019).

Research results from multiple fields (neurobiology, neuroimaging, electrophysiology) have collectively shown that epilepsy is a neural network disease, and its abnormal performance extends greatly outside the original location of the epileptogenic zone, which affects the prognosis of treatment (Gleichgerrcht et al., 2015). Notably, several brain network-based machine learning methods have been proposed for epilepsy analysis or other neurological diseases. These have achieved impressive results (Zhang et al., 2016; Li et al., 2019a; Li et al., 2020b), and are routinely used to aid decision making in epilepsy surgery and other specialties (Cahill et al., 2019; Wang et al., 2019; Shim et al., 2020). A magnetic resonance imaging (MRI) study which used deep learning applied to the whole-brain connectome to determine seizure control after epilepsy surgery attained a positive predictive value (PPV; seizure-freedom) of 88% (Gleichgerrcht et al., 2018). Taking advantage of newer *in vivo* neuroimaging techniques capable of revealing whole-brain metabolic abnormalities by means of positron emission tomography (PET) with 18F-fluorodeoxyglucose (FDG), our group demonstrated that extratemporal metabolic profiles could explain seizure failure after surgery for TLE patients (Tang et al., 2020). Some FDG-PET studies detected multi-scale community structure with different normalization techniques for exhibiting inter-subject FDG-PET brain networks (Sperry et al., 2018). Other studies implemented combined graph theory for measuring aberrant topological patterns in mesial TLE (Wang et al., 2019). Although successful in revealing network abnormalities in clinical groups, these group-level metabolic network analyses sacrifice critical individual-level information. In contrast to earlier work on group-based metabolic patterns, brain network analysis based on graph theory could offer an individualized assessment of metabolic patterns predictive of clinical prognosis (Stam, 2014). The challenge is that aberrant connectivity is variable across individuals, with different patients exhibiting different foci of abnormalities in limbic and extralimbic networks (Gleichgerrcht and Bonilha, 2017). Thus, mapping the brain network in the context of epilepsy could be improved by statistical approaches capable of isolating abnormal individualized patterns in complex data sets.

Inspired by MR-based structural studies using the Kullback-Leibler divergence similarity estimation (KLSE) and the Jensen-Shannon divergence similarity estimation (JSSE) (Tijms et al., 2012b; Kong et al., 2014; Wang et al., 2016b; Li et al., 2021), we analyzed patients with unilateral TLE who had undergone identical surgical resections, and long-term seizure outcomes were analyzed. We opted to apply the JSSE to develop a new analytic methodology for individual-level metabolic brain network construction in FDG-PET imaging and to further provide an ensemble method in predicting the seizure outcomes of TLE patients.

## Materials and Methods

### Participants

We retrospectively studied 128 consecutive patients with a diagnosis of refractory unilateral TLE based on the International League Against Epilepsy (ILAE) criteria (Berg et al., 2010). Comprehensive clinical assessment was performed, including neurological examination, prolonged video electroencephalography (EEG) monitoring, and 3T MRI to confirm either normal MRI or unilateral hippocampal atrophy concordant with the side of seizure onset. Each patient was surgically treated by identical anteromedial temporal resection (AMTR) without extratemporal resections as described by Spencer et al. (1984). All patients underwent FDG-PET brain imaging before surgery. Pathology was assessed from postoperative pathology reports. The determination of postsurgical outcome was based on in-person interviews and patient assessment during clinic follow-up. Patients without 1-year follow-up were excluded from analysis.

Outcome assessments were performed 3 and 12 months after surgery and at yearly intervals thereafter. All patients were interviewed in detail for seizure recurrence, if any, and date of recurrence. Surgical outcomes were classified based on the Engel surgical outcome scale as either seizure free (SZF; Engel class I) or seizure recurrence (SZR; Engel class II through IV) (Engel et al., 2003a; Engel et al., 2003b). Detailed clinical information of the participants can be found in Table 1.

**TABLE 1:** Patient clinical characteristics and surgical outcomes.

Variable	All *n* = 128	SZF *n* = 64	SZR *n* = 64	Stat	*p*-value
Gender (male/female), n	65/63	31/33	34/30	χ2 = 28	0.60
Age, y	26.07 (12.05)	27.41 (11.68)	24.73 (12.35)	t = 1.258	0.21
Age at surgery, y	26.23 (11.97)	27.73 (11.48)	24.75 (12.35)	t = 1.40	0.16
Age at onset*, y	13.48 (10.61)	15.56 (11.95)	11.36 (8.64)	t = 2.26	0.03*
Duration of epilepsy, y	12.68 (8.54)	11.84 (8.48)	13.52 (8.57)	t = 1.11	0.27
Surgical side (L/R), n	73/55	36/28	37/27	χ2 = 0.03	0.86
Hippocampal sclerosis (HS/Non-HS), n	103/25	51/13	52/12	χ2 = 0.05	0.82
Handedness (L/R), n	2/126	2/62	0/64	χ2 = 2.03	0.15
Febrile seizures (with/without), n	18/110	10/54	8/56	χ2 = 0.26	0.61
Brain injury (with/without), n	8/120	2/62	6/58	χ2 = 2.13	0.14
Psychiatric complication (with/without), n	2/126	1/63	1/63	χ2 = 0.0001	0.99
Arua (with/without), n	57/71	29/35	28/36	χ2 = 0.03	0.86
Family history of epilepsy (with/without), n	3/125	1/63	2/62	χ2 = 0.34	0.56
Result of MRI (positive/negative), n	68/60	36/28	32/32	χ2 = 0.50	0.48

Values are shown as mean (SD, standard deviation) unless otherwise specified. *, *p*-value < 0.05.

HS, hippocampal sclerosis; L, left; R, right; MRI, magnetic resonance imaging; SZF, seizure-free (Engel class I); SZR, seizure recurrence (Engel class II, through IV).

All participants provided written informed consent following the Declaration of Helsinki. All aspects of the study were approved by the Studies Institutional Review Board Xiangya Hospital, Central South University.

### FDG-PET Image Acquisition and Processing

FDG-PET was acquired using a Discovery Elite PET/CT scanner (GE Healthcare, Chicago, IL, United States) prior to surgical resection. Images were acquired in 3 dimensions over a 60-min time period, following the scanning protocol described by Tang et al. (2018). Images were reconstructed with an ordered subset expectation maximization algorithm with 6 iterations and 6 subset methods. Individual FDG-PET image volumes were spatially normalized into standard stereotactic Montreal Neurological Institute (MNI) space with linear and nonlinear 3D transformations using statistical parametric mapping software (SPM, Wellcome Department of Cognitive Neurology, London, United Kingdom) on MATLAB (MathWorks, Natick, MA, United States). To facilitate comparison across all participants, the intensity of images was globally normalized. After that, the automated anatomical labeling (AAL) (Tzourio-Mazoyer et al., 2002) atlas was applied to segment the cerebral cortex into 90 regions (45 for each hemisphere without the cerebellum).

### Individual JSSE Metabolic Network Construction

The distribution-divergence–based method has been successfully implemented for individual morphological brain network construction (Tijms et al., 2012a; Kong et al., 2014; Wang et al., 2016a). However, thus far, only a few studies have constructed individual metabolic networks from FDG-PET imaging. We assumed that the FDG-PET signal across brain regions indicates metabolic connections subserving interregional information transfer (Wang et al., 2020). A relatively high resting signal-to-noise FDG-PET signal in a region of interest (ROI) reflects the relative glucose metabolism rate. Thus, this putative relationship offers a plausible approach to characterizing interneuronal information transfer. It is worth noting that most of the existing works have constructed the network using the Kullback-Leibler (KL) divergence (Van Erven and Harremos, 2014):

$$D_{KL}\;\left(\mathbb{P}||\mathbb{Q}\right)=\underset{P}{\int }\left(\mathbb{P}\left(x\right)\log\frac{\mathbb{P}\left(x\right)}{\mathbb{Q}\left(x\right)}\right)dx$$

where ℙ and ℚ represent the probability density functions (PDFs) of voxel intensities in a pair of ROIs, and the KL divergence is asymmetric. In contrast, we instead used the JSSE to capture the statistical relationship of the similarity of cerebral glucose metabolism in any two regions, which could then delineate individual metabolic connections. JS divergence has been successfully used in optimal transport (Lu et al., 2018) and image reconstruction (Jin et al., 2017). The benefits of JSSE are two sides compared to the KL-based methods. Firstly, the range of JS divergence is (0,1), resulting in a more accurate judgment of the similarity. Secondly, JS divergence is symmetric, which makes it easier to portray connections between ROIs. We represented the brain nodes as 90 ROIs from the AAL atlas parcellation for depicting the individual metabolic network. Globally normalized FDG uptake in each ROI was used to generate a region × region correlation matrix (90 × 90) for each participant. The intensity of voxels in each ROI was extracted and used to estimate the PDF of the corresponding ROI with kernel density estimation (Duong, 2007). We then derived the metabolic connections as the Jensen-Shannon (JS) divergence according to the following mathematical equation:

$$D_{JS}\;\left(\mathbb{P}||\mathbb{Q}\right)=\frac{1}{2}\left[D_{KL}\left(\mathbb{P}||Q\right)+D_{KL}\left(\mathbb{Q}||Q\right)\right]$$

where Q=0.5×(ℙ+ℚ) and $D_{KL}\left(\cdot |\cdot \right)$ are the KL-divergence. Here, we used the JS-divergence as a measure of metabolic connectivity to construct the adjacency matrix. In this way, the adjacency matrix describes pairwise metabolic connectivity, where the metabolic connection strength between region i  and j can be represented by the corresponding element in this adjacency matrix.

### Computation of Graph Metrics

We aimed to investigate the altered reconfiguration pattern of the individual brain metabolic connectome for TLE. Based on binary undirected matrices, we systematically analyzed the functional brain network’s global and local properties with the Graph Theoretical Network Analysis Toolbox. Specifically, the global metrics included clustering coefficient ( $C_{p}$ ), characteristic path length ( $L_{p}$ ), normalized clustering coefficient ( γ ), normalized characteristic path length ( λ ), small-world (σ) , global efficiency $\left(E_{global}\right)$ , and modularity score ( Q ) (Newman, 2004). Also, the nodal properties included degree centrality, nodal efficiency, betweenness centrality, shortest path length, and nodal clustering coefficient. The definitions of these measurements can be found in the work of Wang et al. (2015). Both global and nodal graph metrics were applied to characterize the different patterns of connections in the brain network. Notably, we compared the network size at the different sparsity thresholds (of 0.02–0.5, with steps of 0.01), and a sum of 49 values of the corresponding node attributes under the sparsity threshold was obtained. We then took the sum of 49 values for each node [area under the curve (AUC)] as input for the attributes to train the classifier, so there was only one value corresponding to one graph metric.

### Feature Combination and Predicting TLE Outcomes

To accurately differentiate SZF individuals from SZR ones and to develop predictions, we combined information from the connection weights, nodal graph metrics, and global graph metrics. More specifically, we attempted to adopt the kernel combination trick for information combination and used the multi-kernel support vector machine (MK-SVM) for predicting TLE surgical outcomes. The MK-SVM method in this study was conducted as follows. Suppose that there are n training samples with connection values and graph metrics; let $x_{i}^{1}$ , $x_{i}^{2}$ , and $x_{i}^{3}$ represent the connection weight, the graph metrics, and nodal graph metrics of the i -th sample, respectively. With $y_{i}\in \left\{1,-1\right\}$ being the corresponding label, the MK-SVM solves the following primal problem:

$$\underset{W}{\;\min}\frac{1}{2}\overset{3}{\underset{m=1}{\sum }}\beta_{m}∥w^{m}{∥}^{2}+C\overset{n}{\underset{i=1}{\sum }}\xi_{i}$$

$$s.t.\;y_{i}\left(\overset{3}{\underset{m=1}{\sum }}\beta_{m}{\left(w^{m}\right)}^{T}\phi^{m}\left(x_{i}^{m}\right)+b\right)\left(\right)\geq 1-\xi_{i}$$

$$\xi_{i}\geq 0,i=1,2,...,n$$

where $\phi^{m}$ represents the transformation from the original space in m -th data to the Represent Hilbert Kernel Space (RHKS), $\;w^{m}$ represents the hyperplane in RHKS, and $\beta_{m}$ denotes the corresponding combining weight on the m -th attribute. Next, the dual form of MK-SVM can be represented as follows:

$$\underset{\alpha }{\max}\overset{n}{\underset{i=1}{\sum }}\alpha_{i}-\frac{1}{2}\underset{i,j}{\sum }\alpha_{i}\alpha_{j}y_{i}y_{j}\overset{3}{\underset{m=1}{\sum }}\beta_{m}k^{m}\left(x_{i}^{m},x_{j}^{m}\right)\;$$

$$s.t.\overset{n}{\underset{i=1}{\sum }}\alpha_{i}y_{i}=0$$

$$0\leq \alpha_{i}\leq C,i=1,2,\ldots n$$

where $k^{m}\left(x_{i}^{m},x_{j}^{m}\right)=\phi^{m}{\left(x_{i}^{m}\right)}^{T}\phi^{m}\left(x_{j}^{m}\right)$ is the kernel matrix on the m -th data. After we trained the model, we tested the new samples $x=\left\{x_{1},x_{2},\cdots ,x_{M}\right\}$ . The kernel between the new test sample and the i -th training sample on the m -th modality is defined as $k^{m}\left(x_{i}^{m},x^{m}\right)=\phi^{m}{\left(x_{i}^{m}\right)}^{T}\phi^{m}\left(x^{m}\right)$ . Finally, the predictive level based on MK-SVM can be formulated as follows:

$$f\left(x_{1},x_{2},\ldots ,x_{M}\right)=sign\left(\overset{n}{\underset{i=1}{\sum }}y_{i}\alpha_{i}\overset{M}{\underset{m=1}{\sum }}\beta_{m}k^{m}\left(x_{i}^{m},x^{m}\right)+b\right)$$

To illustrate the performance gain of the information combination from different perspectives, such as connection and metrics, we employed the most commonly used and simplest linear kernel, as $k^{m}\left(x_{i}^{m},x_{j}^{m}\right)$ , which is given as follows:

$$k^{m}\left(x_{i}^{m},x_{j}^{m}\right)=x_{i}^{mT}x_{j}^{m}$$

### Feature Selection and Validation

**FIGURE 1:**
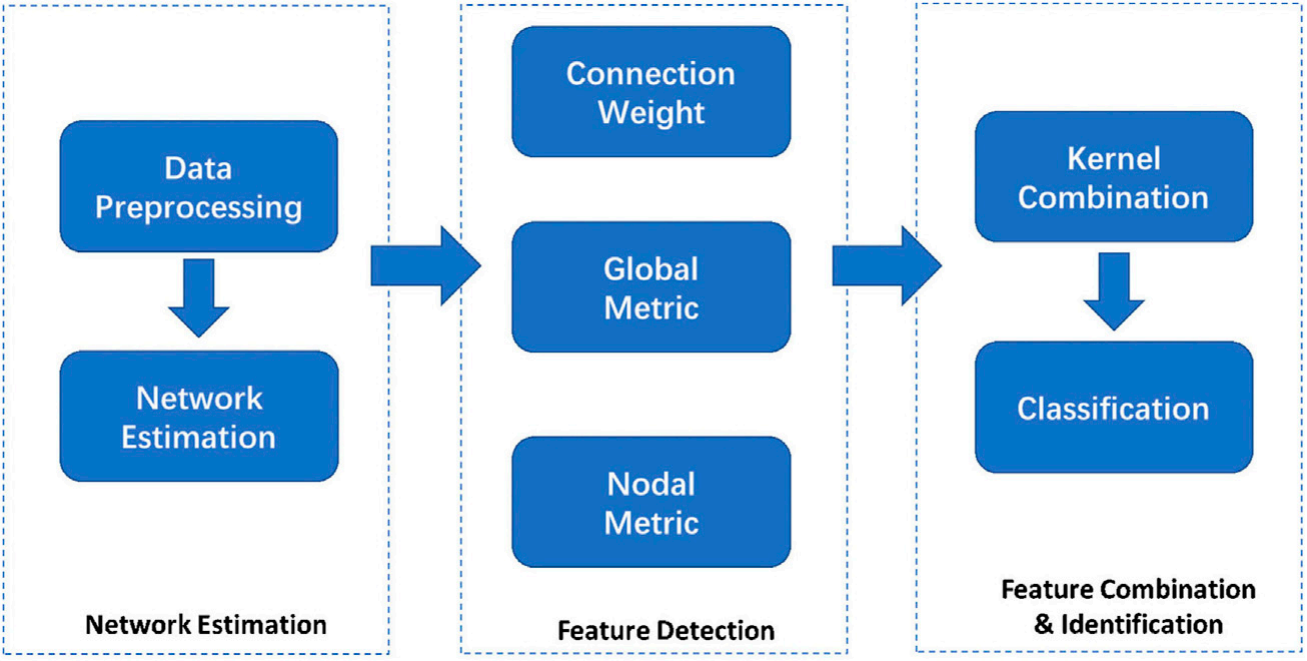
Data-processing and classification procedures employed in our study. Network Estimation: Positron emission tomography (PET) with ^18^F-fluorodeoxyglucose (FDG) imaging of patients were enrolled. Individual FDG-PET image volumes were spatially normalized into standard stereotactic Montreal Neurological Institute (MNI) space with linear and nonlinear 3D transformations using statistical parametric mapping software on MATLAB. Individual’s metabolic brain network was ascertained using the proposed Jensen-Shannon divergence similarity estimation (JSSE) method. Feature Detection: Based on binary undirected matrices, we systematically analyzed the functional brain network’s global and local properties with the Graph Theoretical Network Analysis Toolbox. Also, the nodal properties included degree centrality, nodal efficiency, betweenness centrality, shortest path length, and nodal clustering coefficient. We then took the sum of 49 values for each node as input for the attributes to train the classifier, so there was only one value corresponding to one graph metric. Feature Combination and Identification: We compared the similarity and difference in the JSSE network and its topological measurements between the seizure recurrence and seizure free groups. The two groups were then classified by combining the information from connection and topological metrics, which was conducted by the multiple kernel support vector machine. The validation was performed using the nested leave-one-out cross-validation strategy to confirm the performance of the methods.

To confirm the effectiveness of predicting an individual’s long-term surgical outcomes of TLE, we conducted the nested leave-one-out cross-validation (LOOCV) strategy to verify the performance of the methods due to the small sample size (Li et al., 2020a). in which LOOCV is almost the most strict validation protocol in the machine learning field (Arlot and Celisse, 2010). Specifically, in LOOCV, only one participant was left out for testing while the others were used to train the models and obtain the optimal parameters. For the choice of optimal parameters, an inner LOOCV was conducted on the training data using a grid-search strategy. The range of the hyperparameter C was $2^{-5}\;$ to $2^{5}$ . Meanwhile, to alleviate the interference from the feature selection procedure, we selected the simplest feature selection method (t test with *p* < 0.05) to select the nodal graph metric and the connection weight in our experiment (Li et al., 2019b). All data-processing and classification procedures used in our study are shown in (Figure 1).

### Statistical Analysis

All data were analyzed using SPSS software version 18.0 (IBM Corporation, Armonk, NY, United States). Numerical data are presented as mean with SD. Student’s t test and Pearson’s χ2 test were used for between-group comparisons of continuous variables, as appropriate. To evaluate the classification performance of the information combination methods and the proposed JSSE, we conducted several quantitative measurements, including those determining the accuracy, sensitivity, and specificity. The receiver operating characteristic (ROC) curve and AUC of these methods were also provided. Differences between various AUCs were compared by using a Delong test (Delong et al., 1988). Statistical significance was defined as a *p* value <0.05.

## Results

### Clinical Data

A total of 128 refractory TLE patients (63 females, 65 males; 25.07 ± 12.01 years old; 73 left-TLE, 55 right-TLE) met the inclusion criteria of isolated AMTR with one or more years of follow-up. The median follow-up time was 33 months with a maximum follow-up time of 7 years. The clinical characteristics of the patients are shown in (Table 1). Briefly, 64 of the 128 patients (50%) obtained an Engel class I outcome. There was no significant difference between right TLE (49%) and left TLE (51%) (*p* > 0.05). Regardless of whether there was hippocampal sclerosis (HS) in the postoperative histopathology or MRI, there was no difference in the surgical outcomes (both *p* values > 0.05). According to our statistical estimates, patients with an early age of onset were found to be more likely to relapse (*p* = 0.03).

### Global and Local Graph Metrics of the Metabolic Brain Connectome

The global graph metrics of patients are shown in (Table 2). Ar , Q , $E_{global},E_{local}$ , $\;C_{p}$ , γ , λ , σ , and $S_{r}$ increased, whereas Hr and $L_{p}$ decreased in the SZF group. Statistical analyses revealed that the $A_{r}$ of the SZF group was significantly higher than that of the SZR group.

**TABLE 2:** Global and local graph metrics of the metabolic brain connectome.

Global graph metrics	SZF	SZR
$Ar^{*}$	0.1467 ± 0.02	0.1396 ± 0.02
Q	10.4590 ± 1.03	10.3029 ± 1.37
$H_{r}$	0.0076 ± 0.01	0.0079 ± 0.02
$E_{global}$	0.2232 ± 0.01	0.2228 ± 0.01
$E_{local}$	0.3322 ± 0.01	0.3306 ± 0.01
$C_{p}$	0.2845 ± 0.01	0.2833 ± 0.01
γ	0.6320 ± 0.05	0.6260 ± 0.05
λ	0.5210 ± 0.01	0.5167 ± 0.01
σ	0.5421 ± 0.05	0.5391 ± 0.05
$L_{p}$	1.0451 ± 0.05	1.0453 ± 0.06
$S_{r}$	−0.1499 ± 0.21	−0.2201 ± 0.40

$A_{r}$ , assortativity; $C_{p}$ , clustering coefficient; $E_{global}$ , global efficiency; $E_{local}$ , local efficiency; $H_{r}$ , hierarchy; $L_{p}$ , characteristic path length; Q , modularity score; $S_{r}$ , synchronization; SZF, seizure-free; SZR, seizure-recurrence; γ, normalized clustering coefficient; λ, normalized characteristic path length; σ, small-world. * p-value < 0.05.

### Degree Analysis of the Metabolic Brain Connectome

To investigate the degree distribution of the estimated metabolic brain connectome, we analyzed each node’s mean degree in the SZF and SZR groups. The degree in the IFGoperc.R, ROL. R, IPL. R, and SMG. R tended to be decreased in the SZR group, while the degree in the CAL. L and PCL. L tended to be increased. The 6 significant nodes with the average degree in the SZR and SZF groups are listed in (Table 3).

**TABLE 3:** The 6 significant nodes with the average degree in the SZR and SZF groups.

	SZF	SZR	p-value
IFGoperc.R	17.1175000000000	16.1758208955224	0.0309032527143210
ROL.R	11.0982352941176	9.63402985074627	0.00981312750122977
CAL.L	14.5272058823529	15.4510447761194	0.0398351532853636
IPL.R	14.8452941176471	13.4628358208955	0.0231221430595312
SMG.R	17.2137254901961	15.9493283582090	0.0298336296866740
PCL.L	16.5926470588235	18.0343283582090	0.00882273015415935

SZF, seizure-free; SZR, seizure-recurrence.

The nodes with a degree of SD higher than the mean of the degree of all nodes were identified as degree hub nodes (Rubinov and Sporns, 2010). Here, according to the definition of “hubs,” we identified hub nodes in the SZF and SZR groups separately (Table 4). Comparison of the hub nodes between the two groups in the same modal network clearly revealed that most of them overlapped. Also, it is worth noting that several specific hub nodes corresponded to different groups.

**TABLE 4:** Degree hubs in the SZR and SZF groups.

SZF		SZR	
SFGmed.R	19.2392156862745	SFGmed.L	19.3671641791045
SFGmed.L	18.8848529411765	SFGdor.R	19.1105223880597
SFGdor.R	18.7669607843137	SFGmed.R	19.0319402985075
ORBmid.L	18.0572549019608	PCL.L	18.0343283582090
PCUN.L	17.6072058823530	SOG.L	17.9308955223881
ORBinf.L	17.5039705882353	ORBmid.L	17.9005970149254
MOG.R	17.4164705882353	ORBinf.L	17.8291044776119
SOG.L	17.3668627450981	PreCG.R	17.8264925373134
SMG.R	17.2137254901961	PCUN.L	17.3124626865672
IFGoperc.R	17.1175000000000	SOG.R	17.0566417910448
ITG.R	17.0490686274510	PoCG.L	16.8581343283582
PreCG.R	17.0400000000000	—	—
IOG.R	16.9177450980392	—	—
MTG.L	16.8850980392157	—	—
SOG.R	16.8262745098039	—	—
PoCG.L	16.7069607843137	—	—

ZF, seizure-free; SZR, seizure-recurrence.

### Betweenness Analysis of the Metabolic Brain Connectome

We also investigated the betweenness distribution of the estimated metabolic brain connectome of the SZR and SZF groups. The results showed that betweenness in ORBsup.R and IOG. R tended to be decreased in the SZR group, while betweenness in PreCG.R, IOG. R, PoCG.R, PCL. L, and PCL. R tended to be increased. The 6 significant nodes with the average betweenness in the SZR and SZF groups are listed in (Table 5). We also identified betweenness hub nodes of SZR patients and SZF patients (Table 6). Comparison of the betweenness hub nodes between the SZR group and SZF group in the same modal network revealed that several specific hub nodes corresponded to different groups.

**TABLE 5:** The 6 significant nodes with the average betweenness in the SZR and SZF groups.

	SZF	SZR	*p*-value
PreCG.R	15.3758228782319	18.3473121940106	0.0361029392125115
ORBsup.R	34.6001343347223	25.4420785472724	0.0352784404129758
IOG.R	21.3237440032479	16.3262297494005	0.0140831513048402
PoCG.R	9.34738257361109	11.8143499562684	0.0319301784701826
PCL.L	21.4726364317984	28.4648712016924	0.00712058470205053
PCL.R	16.9695647930214	23.4512654507503	0.0121138051983782

SZF, seizure-free; SZR, seizure-recurrence.

**TABLE 6:** Betweenness hubs in the SZR and SZF groups.

SZF		SZR	
PUT.L	69.3871776909212	PUT.L	70.4504461037482
PUT.R	48.9657146312205	PUT.R	54.3317298368621
ITG.R	45.3484691576060	ORBinf.L	45.2615602093781
DCG.L	45.0242583545777	ITG.R	41.1503972000794
ORBinf.L	42.5620251509945	DCG.L	40.1726525213182
ITG.L	41.1909171341282	ORBmid.R	38.5886577741851
ORBmid.R	38.0795677180871	ITG.L	32.9533102874909
ORBsup.R	34.6001343347223	INS.L	31.4995732390936
DCG.R	32.3583202714907	DCG.R	30.6233992842629
INS.R	32.1463842421356	SFGdor.R	30.2490589173754
SFGmed.R	31.9942448706237	—	—
THA.R	31.7894415757695	—	—
ORBsup.L	31.2743927209494	—	—

SZF, seizure-free; SZR, seizure-recurrence.

### Classification Results

**FIGURE 2:**
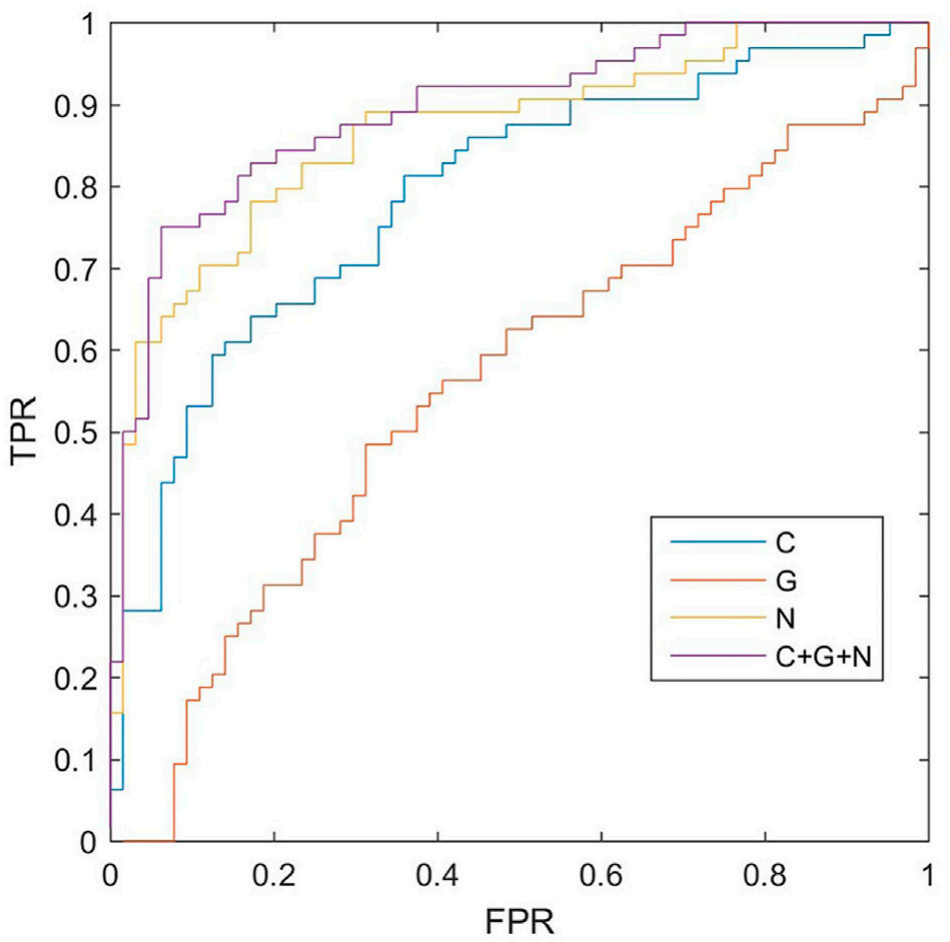
The receiver operating characteristic (ROC) curve results of different methods. The ROC curve result indicate that the performances of information combination results are superior to those of single kernel methods. Additionally, the connection weights (C) + global metrics (G) + nodal metrics (N) method achieved the outperforming of results in all four measurements, demonstrating its effectiveness. Furthermore, according to DeLong’s nonparametric statistical significance test, the proposed C + G + N method (purple line) was found to be significantly superior to C, G, and N under 95% confidence intervals with *p* values equal to 2 × 10^−5^, 6 × 10^−8^, and 0.045, respectively. These aberrant functional network measures exhibited ideal classification performance in predicting seizure free individuals from seizure recurrence ones at a sensitivity of 75.00%, a specificity of 92.79%, and an accuracy of 83.59%.

To evaluate the classification performance of the information combination methods and the proposed JSSE, we also reported the single kernel SVM classification result based on the connection weights (C), global metrics (G), and nodal metrics (N). The results are shown in (Table 7). The ROC curve result is shown in (Figure 2), indicating that the performances of information combination results are superior to those of single kernel methods, thus supporting the rationality of the proposed method. Additionally, the C + G + N method achieved the outperforming of results in all four measurements, demonstrating its effectiveness. Furthermore, according to DeLong’s nonparametric statistical significance test (Zhang et al., 2016), the proposed C + G + N methods were found to be significantly superior to Connection, Global, and Nodal under 95% confidence intervals with *p* values equal to 2 $\times 10^{-5}$ , 6 $\times 10^{-8}$ , and 0.045, respectively. The superior performance illustrated that the information combination scheme could effectively improve the classification performance. These aberrant functional network measures exhibited ideal classification performance in predicting SZF individuals from SZR ones at a sensitivity of 75.00%, a specificity of 92.79%, and an accuracy of 83.59%.

**TABLE 7:** Classification performance corresponding to different methods.

Method	Accuracy	Sensitivity	Specificity	AUC
Connection (C)	73.44	64.06	82.81	0.9058
Global (G)	56.25	57.81	54.69	0.7242
Nodal (N)	78.91	75.00	82.81	0.8354
C + G + N	83.59	75.00	92.19	0.9571

C + G + N methods are significantly superior to Connection, Global, and Nodal under 95% confidence interval with *p*-value equals to 2 $\times 10^{-5}$ , 6 $\times 10^{-8}$ and 0.045 respectively.

### Consensus of Significant Metabolic Connections

**FIGURE 3:**
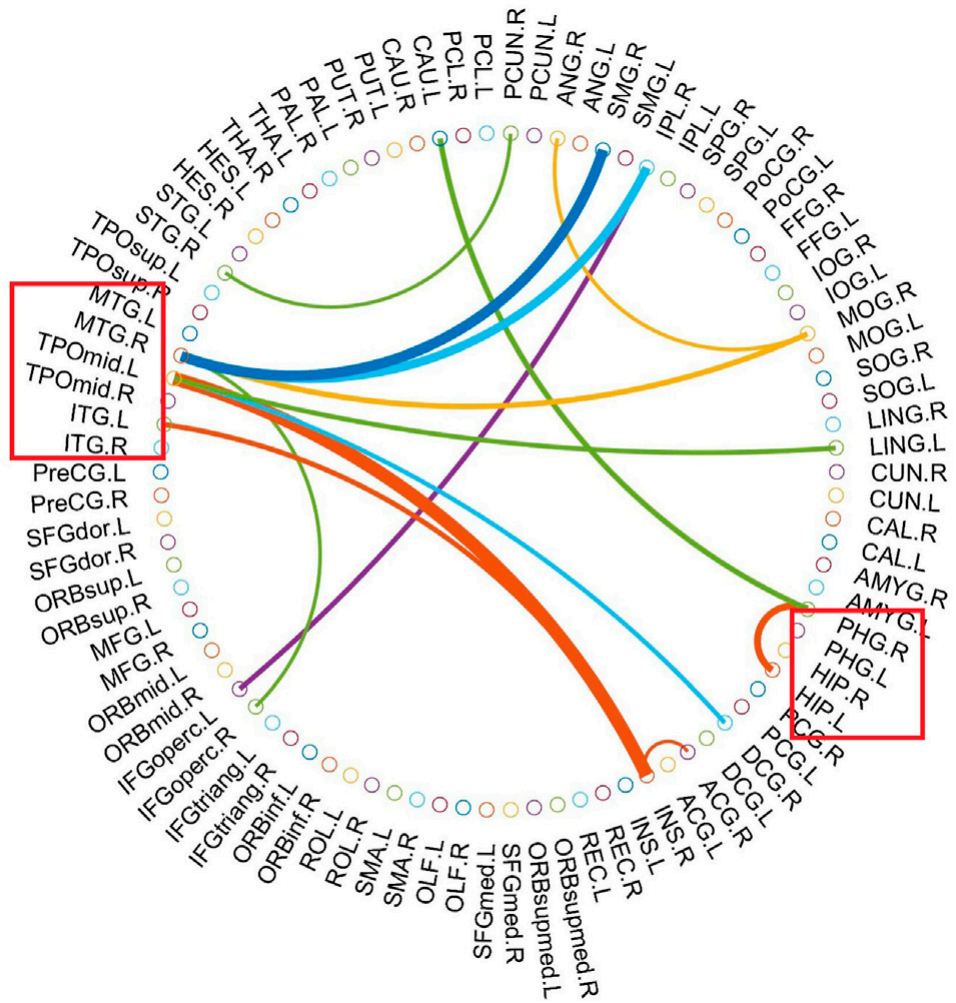
The most consensus connections. The most consensus connections mapped on the International Consortium for Brain Mapping (ICBM) 152 template using the BrainNet Viewer software package http://nitrc.org/projects/bnv/and circularGraph, shared by Paul Kassebaumb http://www.mathworks.com/matlabcentral/fileexchange/48576-circulargraph). The red box represents the temporal lobe containment area (HIP, hippocampus; ITG, inferior temporal gyrus; MTG, middle temporal gyrus; PHG, parahippocampal gyrus; TPOmid, middle temporal gyrus). We selected the consensus connections with a *p* value <0.05 in each loop. A total of 45 consensus connections are shown. In exploring the consensus significant metabolic connections, we observed that the most involved metabolic motor networks were the INS-TPOmid.L, MTG. R-SMG. R, and MTG. R-IPL.R pathways between the seizure recurrence and seizure free groups, and yielded further detailed individual pathological connectivity in the PHG. R-CAU.L, PHG. R-HIP.L, TPOmid.L-LING.R, TPOmid.L-DCG.R, MOG. R-MTG.R, MOG. R-ANG.R, and IPL. R-IFGoperc.L pathways.

As mentioned above, we selected the consensus connections with a *p* value <0.05 in each loop. A total of 45 consensus connections are shown in (Figure 3). In exploring the consensus significant metabolic connections, we observed that the most involved metabolic motor networks were the INS-TPOmid.L, MTG. R-SMG. R, and MTG. R-IPL.R pathways between the two groups, which was consistent with the results of the typical group-level method, and yielded further detailed individual pathological connectivity in the PHG. R-CAU.L, PHG. R-HIP.L, TPOmid.L-LING.R, TPOmid.L-DCG.R, MOG. R-MTG.R, MOG. R-ANG.R, and IPL. R-IFGoperc.L pathways.

## Discussion

TLE is the most common drug-resistant epilepsy in adults and is traditionally associated with HS, a lesion affecting the hippocampus and adjacent mesial structures (Blumcke et al., 2013). Evidence from neuroimaging and neuroelectrophysiology has consistently demonstrated that epilepsy is a disease of abnormal networks, with changes occurring well beyond the focus of ictogenesis. These findings have been corroborated by mounting neuroimaging evidence suggesting the presence of diffuse grey and white matter abnormalities beyond the mesial temporal lobe that affect a distributed network of cortical and subcortical structures as well as their connections (Nikolin et al., 1984; Biller et al., 1986; Lin et al., 2007). Thus, the architecture of a patient’s brain network may have the answer to a critical question in the field of epilepsy: Why do some TLE patients, despite being deemed optimal surgical candidates, fail to achieve seizure control after epilepsy surgery? To address this issue, we investigated a larger group of patients with a homogeneous clinicopathological syndrome undergoing standard AMTR for TLE at a single center with a long postoperative follow-up.

We developed an individual-level metabolic network construction approach for presurgical FDG-PET imaging and applied it to the task of predicting individual seizure outcomes after epilepsy surgery. The classification accuracy of MK-SVM *via* combining the information from connection and topological metrics showed a sensitivity of 75.00%, a specificity of 92.79%, and an accuracy of 83.59%. Our approach may further the mechanistic understanding of seizure recurrence by identifying abnormal graph metrics of the metabolic brain connectome. If we can identify the specific degree and betweenness analysis of individual metabolic brain connectome abnormalities associated with seizure outcomes, we can potentially promote earlier referral to epilepsy surgery in patients deemed to be favorable candidates. This, in turn, would likely lead to a reduced psychosocial burden and improved quality of life.

Some studies have investigated surgical outcomes by considering the brain as a network of connected regions. Network measures that have been found to be altered in TLE include the clustering coefficient of a region, which captures the connectedness of neighbors of a region (Bernhardt et al., 2011). Furthermore, regression analysis and machine learning approaches have also been applied to brain networks of TLE to relate them to surgical outcomes (Bonilha et al., 2013; Bonilha et al., 2015; Ji et al., 2015; Munsell et al., 2015). Until now, most FDG-PET imaging studies of metabolic networks have used group-level analyses, which potentially sacrifice or obscure salient individual differences within a group; in contrast, our novel JSSE approach offers individual risk estimation. The JSSE or KLSE approach can perhaps be successfully applied in individual structural MR-based analyses (Tijms et al., 2012b; Kong et al., 2014; Wang et al., 2016b; Li et al., 2021), but have not yet been applied in metabolic maps in epilepsy. Based on the JSSE calculation of relative entropy, it can quantify the interregional metabolic interactions for the construction of an individual’s metabolic brain network. The putative association of FDG metabolism with afferent synaptic activity suggests that the various elements of the connectome are metabolically coupled (Raichle and Mintun, 2006). In the current state of FDG-PET, the intraregional similarity calculated according to the JSSE is a surrogate measure of the metabolic connectivity between brain regions.

Recapitulating previous studies on seizure outcome prediction after TLE epilepsy surgery, these works indeed have successfully differentiated seizure freedom vs. recurrence (Tellez-Zenteno et al., 2005; Bernhardt et al., 2011; Bonilha et al., 2013; Jehi et al., 2015b; Bonilha et al., 2015; Gleichgerrcht et al., 2015; Gleichgerrcht et al., 2018; Cahill et al., 2019; Wang et al., 2019; Shim et al., 2020). Nevertheless, such approaches may not depict individual pathophysiological details or downstream clinical therapeutic strategies, whereas our novel JSSE approach can provide individual risk estimation. The symptoms of many neurological and psychiatric diseases are mappable to specific functional networks of interconnected brain regions. Based on a novel *in vivo* approach that combined FDG-PET metabolic connectivity and physical distance between cortical areas, we were able to parameterize the balance between short- and long-range functional connections. Comparing SZR to SZF, we observed marked degree reductions in IFGoperc.R, ROL. R, IPL. R, and SMG. R; and betweenness reductions in ORBsup.R and IOG. R; meanwhile, CAL. L and PCL. L tended to be increased from degree analysis; and PreCG.R, IOG. R, PoCG.R, PCL. L, and PCL. R tended to be increased from betweenness analysis. Exploring consensus significant metabolic connections, we observed that the most involved metabolic motor networks were the INS-TPOmid.L, MTG. R-SMG. R, and MTG. R-IPL.R pathways between the two groups, which was similar to findings of the typical group-level method, and yielded further detailed individual pathological connectivity in the PHG. R-CAU.L, PHG. R-HIP.L, TPOmid.L-LING.R, TPOmid.L-DCG.R, MOG. R-MTG.R, MOG. R-ANG.R, and IPL. R-IFGoperc.L pathways. Collectively, our findings provide a new individualized metabolic JSSE network analysis capable of revealing subtle deviations in metabolic connectivity as a potential disease mechanism of TLE and which supports the promising clinical benefits of combining connectomic data with physically grounded information.

Most studies have focused on the identification of isolated outcome predictors, and a few studies have associated various combinations of outcome predictors with postoperative seizure control (Lee et al., 2005; Kumar et al., 2013). However, it is difficult to objectively combine a patient’s complex clinical characteristics and frequent multiple contradictory outcome predictors into a single comprehensive validated risk assessment measure (Jehi et al., 2015a; Jehi et al., 2015b; Keezer et al., 2015). This absence leads to uncertainty during presurgical counseling because most candidates for surgery do not fall into clear categories with all negative or all positive outcome predictors (Jehi et al., 2015b). Related studies have shown that some predictors work only for specific patient groups (Kumar et al., 2013). To compare the accuracy provided by our classification approach vs. clinical variables, we performed a discriminant function analysis that included 14 clinical variables (featured in Table 1). We also further explored the surgical outcomes in TLE with specific combinations of clinical characteristics. Only age at onset was found to correlate with surgical results, but the prognostic value of this indicator has been shown to be limited (Lee et al., 2005).

The decision to undergo surgery for epilepsy is complex and variable, and depends on multiple factors, including the patient’s baseline disease burden and overall clinical picture, not solely on the chance that seizures can be alleviated. Our approach was not meant to replace clinical judgment, but rather to enrich it by providing an objective and quantifiable estimate for a single key decision-driving factor (postoperative seizure outcome). In our previous study, in addition to discovering the number of metabolic abnormalities in the extratemporal area associated with surgical failure, we also described special patterns associated with the failure of TLE surgery (Tang et al., 2020), which spanned from the frontal and parietal regions to the occipital and contralateral regions, and some of which are consistent with electroclinical patterns corresponding to anterior and posterior spread (Chassoux et al., 2016). Our present brain connectome approach can measure local network properties and the entire network, and combined with MK-SVM, can provide powerful identification of the salient properties predictive of the surgical outcomes in SZF and SZR patients (accuracy of 83.59%). In metabolic brain connectome regard, our main finding was that SZR entails low of assortativity of the global and local graph metrics and a loss of connectivity between these modules. With the progression of TLE, these affected brain regions fail to metabolically compensate in TLE patients who inevitably undergo further disturbances. This latter result is consistent with previous studies showing the failure of network components early in the epilepsy process that lead to temporal lobe surgery failures (Ji et al., 2015; Giulioni et al., 2016; van den Heuvel and Sporns, 2019). Furthermore, we tested for a consensus of significant metabolic connections between SZR and SZF TLE patients, as summarized in Figure 3. The results accord with group-level studies showing analogous network changes in surgical failure (Barba et al., 2016; Bernhardt et al., 2016; Chassoux et al., 2017; Gleichgerrcht et al., 2018; Tang et al., 2020).

There are several limitations in this study. First, we did not include other important outcomes of interest after epilepsy surgery, such as quality of life, mood, and psychosocial functioning. Furthermore, this was a retrospective analysis and all patients underwent FDG-PET scanning, leading to a low proportion of class I Engel outcomes (50%) (Engel et al., 2003a; Tellez-Zenteno et al., 2005). Patients who had not undergone FDG-PET imaging were excluded, which might have led to some selection and ascertainment biases. Finally, preoperative video EEG data, structural MRI neuroimaging, and the results of more sophisticated diagnostic tests, such as single photon–emission computed tomography and invasive EEG were not analyzed in study patients who failed TLE surgery.

## Conclusion

This study presents an advanced connectome analysis of FDG-PET images based on a novel application of JSSE entropy measures, which had not been previously applied to the task of metabolic connectome analysis. Our findings shed new light on the network abnormality underlying TLE and, importantly, provide novel understandings and additional evidence in furthering the mechanism research in TLE. Furthermore, our results demonstrate that information combinations from different views can achieve ideal performance in predicting an individual TLE patient’s long-term surgical outcome.

## Data Availability

The original contributions presented in the study are included in the article/Supplementary Material, further inquiries can be directed to the corresponding author.
